# Genomic Characterization of Concurrent Alterations in Non-Small Cell Lung Cancer (NSCLC) Harboring Actionable Mutations

**DOI:** 10.3390/cancers13092172

**Published:** 2021-04-30

**Authors:** Antonio Passaro, Ilaria Attili, Alessandra Rappa, Davide Vacirca, Alberto Ranghiero, Caterina Fumagalli, Juliana Guarize, Lorenzo Spaggiari, Filippo de Marinis, Massimo Barberis, Elena Guerini-Rocco

**Affiliations:** 1 Division of Thoracic Oncology, IEO, European Institute of Oncology, IRCCS, 20141 Milan, Italy; Ilaria.attili@ieo.it (I.A.); filippo.demarinis@ieo.it (F.d.M.); 2 Division of Pathology and Laboratory Medicine, IEO, European Institute of Oncology, IRCCS, 20141 Milan, Italy; alessandra.rappa@ieo.it (A.R.); davide.vacirca@ieo.it (D.V.); alberto.ranghiero@ieo.it (A.R.); caterina.fumagalli@ieo.it (C.F.); massimo.barberis@ieo.it (M.B.); elena.guerinirocco@ieo.it (E.G.-R.); 3 Department of Thoracic Surgery, IEO, European Institute of Oncology, IRCCS, 20141 Milan, Italy; juliana.guarize@ieo.it (J.G.); lorenzo.spaggiari@ieo.it (L.S.); 4 Department of Oncology and Hemato-Oncology, University of Milan, 20122 Milan, Italy

**Keywords:** predictive biomarker, next generation sequencing, co-mutation, EGFR, MET, KRAS, BRAF, ALK, ERBB2, RET

## Abstract

**Simple Summary:**

Genomic driver alterations with potential impact on prognosis and treatment response are increasing in non-small cell lung cancer (NSCLC). However, few comprehensive data are available on tumor heterogeneity in all these specific subgroups. We conducted this research with the aim to describe specific molecular co-mutation patterns occurring across the main actionable gene subgroups of NSCLC. The findings from the current research are proposed as a backbone in the knowledge of molecular heterogeneity as analyzed by comprehensive genomic profiling in NSCLCs with driver gene alterations, at the basis of subsequent evaluations related to clinical outcomes.

**Abstract:**

An increasing number of driver genomic alterations with potential targeted treatments have been identified in non-small cell lung cancer (NSCLC). Much less is known about the incidence and different distribution of concurrent alterations, as identified by comprehensive genomic profiling in oncogene-addicted NSCLCs. Genomic data from advanced NSCLC consecutively analyzed using a broad next-generation sequencing panel were retrospectively collected. Tumors harboring at least one main actionable gene alteration were categorized according to the presence/absence of concurrent genomic aberrations, to evaluate different patterns among the main oncogene-addicted NSCLCs. Three-hundred-nine actionable gene alterations were identified in 284 advanced NSCLC patients during the study period. Twenty-five tumor samples (8%) displayed concurrent alterations in actionable genes. Co-occurrences involving any pathogenic variant or copy number variation (CNV) were identified in 82.8% of cases. Overall, statistically significant differences in the number of concurrent alterations, and the distribution of *TP53*, *STK11*, cyclines and receptor tyrosin kinase (RTK) aberrations were observed across the eight actionable gene groups. NGS analyses of oncogene-addicted NSCLCs showed a different distribution and pattern of co-alteration profiles. Further investigations are needed to evaluate the prognostic and treatment-related impact of these concurrent alterations, hooked to the main gene aberrations.

## 1. Introduction

The presence of oncogene drivers in non-small cell lung cancer (NSCLC) identifies a subgroup of patients with potential survival benefit from targeted therapies in the advanced and metastatic setting. Patients harboring actionable gene mutations and treated with a targeted agent showed a significant improvement in efficacy endpoints (e.g., overall response rate, progression-free survival and overall survival) and decreased risk of death, compared to those not receiving targeted treatment and to those without oncogene addiction [1]. Since the identification of activating oncogenic mutations in the tyrosine kinase domain of epidermal growth factor receptor (EGFR) in 2004 [2], an increasing number of activating oncogene alterations have been identified in NSCLC, with a related growing knowledge of their impact on treatment selection and survival [3,4]. In the latest years, several tyrosine kinase inhibitors have been developed to target different novel driver gene alterations other than *EGFR*, *ALK*, *ROS-1* and *BRAF*, including *RET*, *MET* and *ERBB2*, and some of them have already received approval by the Food and Drug Administration (FDA) and European Medicines Agency (EMA) for the treatment of advanced NSCLC. Despite the high efficacy of these target agents, all patients develop disease progression due to primary or acquired resistance mechanisms [5,6]. To date, a deeper understanding of individual tumor heterogeneity and evolutionary pressures related to the different therapies is highly useful to address and define future potential treatment strategies for patients with oncogene-addicted NSCLCs [7,8,9].

Relevant improvements in treatment strategies have been reached in parallel with significant advancements in molecular diagnostics. Next-generation sequencing (NGS) assays allow us to simultaneously detect multiple alterations in different genes, including but not limited to the known genetic drivers. Therefore, they allow insights into tumor molecular heterogeneity by evaluating the presence of co-occurring genomic alterations, which might be at the basis of clinical heterogeneity and the variable response to anticancer treatments within oncogenic-driven subgroups. Interest is growing towards the possibility to define diverse NSCLC phenotypes through the identification of different co-mutational patterns [10,11], and a negative prognostic role has been described for *TP53* and *LKB1* (or *STK11*) in *EGFR* and *KRAS* mutant lung cancer, respectively [12,13,14,15,16,17]. Additional gene alterations, including cycline-related genes and *PIK3CA*, have also been initially described as singular determinants of molecular diversity potentially impacting clinical results in NSCLCs with driver mutations [14,18,19]. However, few data are available regarding comprehensive co-mutation patterns, in particular in the less frequently detected oncogene-addicted NSCLCs (i.e., *ALK*, *ROS1* or *RET* rearrangements, *BRAF* or *ERBB2* mutations).

In a previous report, we described the feasibility and potential impact of genomic profiling in lung cancer using a 22-gene-based NGS assay [20], showing that 82.4% of non-squamous NSCLC harbored at least one molecular alteration, while 63.6% carried clinically relevant molecular aberrations.

This work aims to deeply investigate the comprehensive molecular results obtained by wider genomic profiling of lung cancers, covering fusion genes and copy number variants (CNVs), in addition to gene mutations, to evaluate the patterns of concurrent alterations across the main actionable gene subgroups and to improve the molecular and clinical understanding of oncogene-addicted non-small cell lung cancer.

## 2. Materials and Methods

We retrospectively collected tumor genomic data from patients with advanced NSCLC consecutively referred to the European Institute of Oncology from January 2018 to September 2020. The study population included treatment-naïve patients diagnosed at our facility and patients who were referred to our center after being diagnosed with NSCLC in other institutions in the absence of a molecular profile. For the purpose of this study, only tumors with complete molecular reports obtained from broad next-generation sequencing panel analysis were considered. Additional results from fluorescence in situ hybridization (FISH) testing were reviewed, whenever available. All information regarding human material was managed using anonymous numerical codes, and all samples were handled in compliance with the Helsinki Declaration.

Molecular analysis was performed with a 161-gene NGS assay (Oncomine Comprehensive Assay v.3; ThermoFisher Scientific, Waltham, MA, USA), according to the manufacturer’s instructions. Briefly, 10 ng of DNA and RNA extracted from available representative tumor samples (formalin-fixed paraffin-embedded (FFPE) tissue blocks, cytoblocks or smears) were used for the library and template preparation on the Ion Chef System (ThermoFisher Scientific, Waltham, MA, USA). The sequencing run was performed on the Ion S5 System (ThermoFisher Scientific, Waltham, MA, USA) and data were analyzed with the Ion Reporter Analysis Software (ThermoFisher Scientific, Waltham, MA, USA). Only mutations with a variant allele frequency (VAF) equal/superior to 5%, adequate quality metrics and annotated as pathogenic/likely pathogenic in cancer gene mutation databases (Catalogue of Somatic Mutations in Cancer (COSMIC), cBioPortal for Cancer Genomics, ClinVar–NCBI–NIH) were recorded. Copy number gains (CNG) were evaluated only for samples with a median of the absolute values of all pairwise difference (MAPD) <0.5 [21]. Variants of unknown significance (VUS) and variants classified as polymorphism, benign, likely benign or neutral were not considered for the purpose of our study.

FISH analyses were performed to confirm the presence of gene rearrangements and copy number gain detected by NGS analysis using a dual-color probe (IQFISH Break Apart Probe and MET IQFISH Probe with CEP7, respectively; Agilent Technologies, Santa Clara, California, USA). The gene copy number (GCN) cutoff of 6 was used to define *MET*-amplified cases for this study, either by FISH or by NGS.

All patients with any of the acknowledged actionable gene alterations in NSCLC were included in the study analysis. Specifically, the eight evaluated actionable gene alterations were *EGFR*, *KRAS* and *BRAF* mutations, *ALK*, *ROS1* and *RET* rearrangements, *MET* deregulation and *ERBB2* insertions. Due to the different clinical behavior and response to specific treatments, *EGFR* mutations were further subclassified into common, sensitive—exon 19 deletions or exon 21 L858R point mutation—and uncommon mutations. Similarly, *BRAF* mutations were subclassified into V600 and non-V600 point mutations, *KRAS* into G12C and non-G12C mutations and *MET* into exon 14 skipping mutations and amplifications.

The identified concurrent alterations were grouped into 9 categories according to gene functions (Table S1): TP53, STK11, DNA repair pathway, beta-catenin, MYC pathway, PI3K pathway, cycline pathways, receptor tyrosine kinases (RTKs) and others.

Variables were presented by using the median value for continuous variables and percentages (numbers) for categorical variables.

Pairwise comparisons using t-tests with pooled standard deviation (SD) were used to evaluate the differences in the co-occurrence patterns according to the eight main driver gene groups.

Odds ratio (OR), calculated using exact methods (mid-*p* and Fisher) and normal approximation (Wald), was used to evaluate the association between genes and concurrent alterations. Relative risk (RR) was used instead of OR for cases with a 0 value as the reference level.

Statistical significance level was set at *p* < 0.05 for all tests. All statistical analyses were performed with RStudio (RStudio: Integrated Development for R. RStudio, Inc., Boston, MA). Co-occurrence of selected gene alterations was also evaluated using the mutual exclusivity analysis of the cBioPortal bioinformatics tool [22,23,24].

## 3. Results

### 3.1. Distribution of Actionable Gene Mutations in the Study Population

Overall, among 392 patients, we identified 284 cases (72%) with advanced NSCLC harboring at least one actionable gene alteration and complete molecular data as tested with the NGS panel. A total of 309 actionable alterations were identified: 135 (43.7%) KRAS mutations (*n* = 49 G12C and *n* = 86 non-G12C point mutations), 62 (20%) EGFR mutations (*n* = 50 common and *n* = 12 uncommon mutations), 29 (9.4%) MET alterations (*n* = 6 exon 14 skipping and *n* = 23 gene amplification), 41 (13.3%) ALK rearrangements, 10 (3.2%) BRAF mutations (*n* = 3 V600 and *n* = 7 non-V600 point mutations), 12 (3.9%) ROS1 rearrangements, 16 (5.2%) RET rearrangements and 4 (1.3%) ERBB2 exon 20 mutations (Table 1; Table S2).

### 3.2. Frequency and Distribution of Concurrent Genomic Aberrations across the Actionable Gene Subgroups

**Figure 1:**
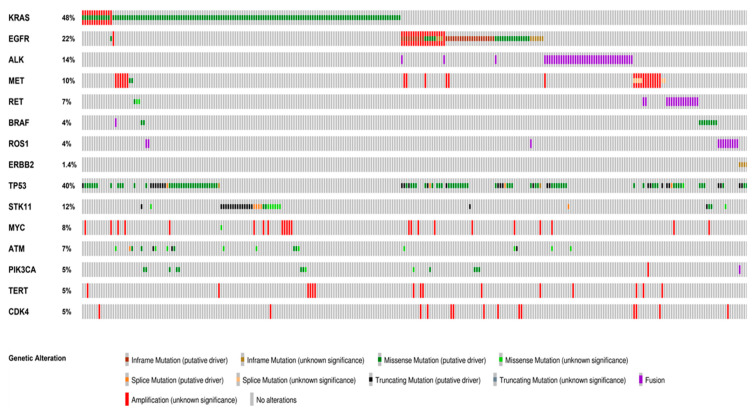
Distribution and co-occurrence of actionable and concurrent gene alterations. Oncoprint plots showing the distribution of actionable gene alterations, concurrent actionable alterations and other co-occuring gene alterations identified in more than 5% of the cases. The type of alteration is color-coded according to the legend below. Co-occurrences of actionable gene alterations included *MET* amplification, *KRAS* and *EGFR* mutations. The most frequent concurrent alterations were *TP53* and *STK11* mutations. Oncoprinter tool—cBioportal (https://www.cbioportal.org/oncoprinter, accessed on 10 March 2021).

Overall, the presence of concurrent pathogenic or likely pathogenic alterations was identified in 256 out of the 309 (82.8%) actionable gene aberrations, including cases with concurrent actionable alterations. Concurrent actionable gene alterations occurred in 25 (8%) of the 284 patients, including MET gene amplification (17.68%), KRAS (10.40%) and EGFR (9.36%) mutations (Figure 1; Figure S1).

**Figure 2:**
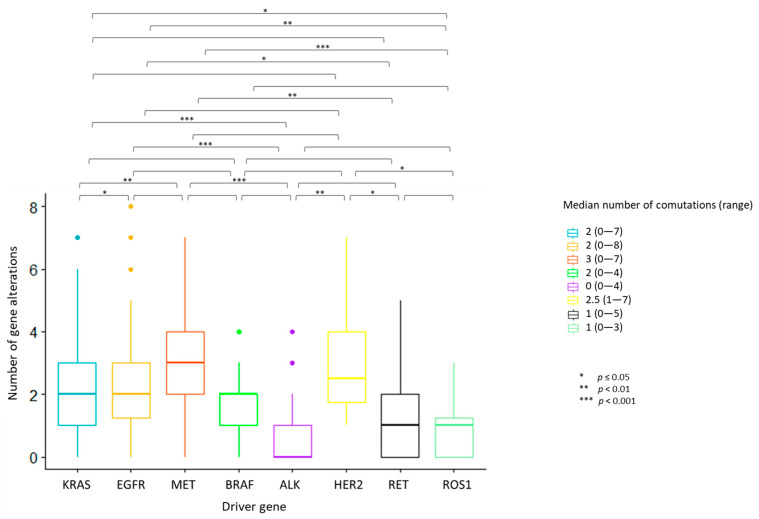
Number of concurrent gene alterations across the eight actionable gene groups. Each bar represents the number of concurrent gene alterations across the eight actionable gene groups. The median number and the range of concurrent gene alterations identified in each actionable gene group are reported in the legend on the right. Statistically significant differences in the distribution of the median number of total concurrent alterations were seen across the actionable gene groups, with the highest median number of concurrent alterations identified in MET deregulated tumors. ALK, ROS1 and RET-positive tumors displayed the lowest median number of co-occurrences. One-way ANOVA test for variance analysis. * *p* ≤ 0.05; ** *p* < 0.01; *** *p* < 0.001

The median number of overall observed genomic co-alterations (pathogenic/likely pathogenic mutations or CNGs) was two (range 0–8). One-way ANOVA test for variance analysis highlighted a statistically significant difference in the distribution of the median number of total concurrent alterations across the eight actionable gene groups (*p* < 0.001). Cases with ALK, ROS1 and RET rearrangements showed the lowest median number of concurrent alterations; MET deregulated tumors displayed the highest median number of co-occurrences (Figure 2). Multiple pairwise comparisons revealed differences between specific pairs of groups (Figure 2; Figure S2 and File S1).

ALK-rearranged tumors were significantly less likely to harbor co-mutations as compared to cases with KRAS (OR: 0.24, 95% CI 0.11–0.49, *p* = 0.00019), EGFR (OR: 0.15, 95% CI 0.06–0.38, *p* < 0.0001), MET (OR: 0.13, 95% CI 0.03–0.42, *p* = 0.0004) and BRAF (OR: 0.10, 95% CI 0.004–0.62, *p* = 0.014) alterations (Figure S3A). Similarly, a lower frequency of concurrent CNVs was detected in ALK-positive cases compared to KRAS, EGFR and MET-altered tumors (OR: 0.24, 95% CI 0.076–0.595; OR: 0.10, 95% CI 0.03–0.28; OR: 0.14, 95% CI 0.04–0.43, respectively). Tumors with EGFR mutations harbored more frequently concurrent CNVs than those with KRAS mutations (OR: 2.27, 95% CI 1.23–4.23) and RET and ROS1-rearranged tumors (OR: 5.67, 95% CI 1.61–28; OR: 13.19, 95% CI 2.31–339, respectively). ROS1-positive cases were less likely to present with CNVs compared to MET-deregulated patients (OR: 0.099, 95% CI 0.004–0.63) (Figure S3B). No significant differences in the occurrence of co-mutations or CNGs were observed within actionable gene subclasses, except for MET-deregulated tumors, where a higher frequency of co-mutations was found in MET-amplified as compared to MET exon 14 skipping cases (100 vs. 33.3%, *p* < 0.001) (Table 1).

### 3.3. Distinctive Patterns of Concurrent Alterations in the Actionable Gene Subgroups

**Figure 3:**
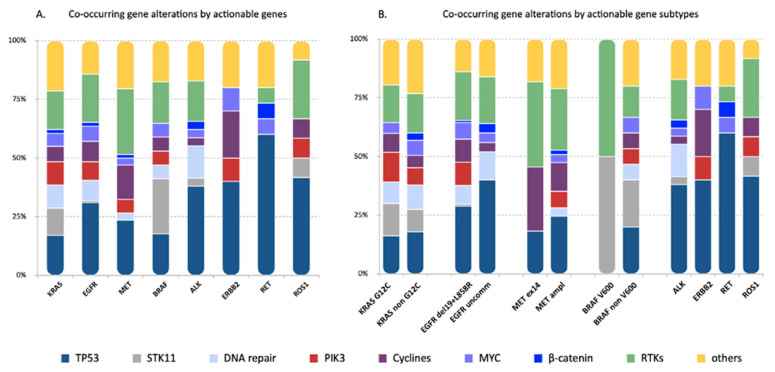
Genes and/or pathways affected by the concurrent alterations identified in the eight actionable gene groups. Each bar represents one actionable gene group. The frequency of genes and/or pathways affected by the concurrent alterations were reported for each actionable gene group (**A**) and actionable gene subclass (**B**) and color-coded according to the legend below. Distinctive concurrent gene and/or pathway alterations were identified in the different actionable gene groups. In particular, *TP53* mutations were more frequently detected in EGFR, ERBB2 and RET-positive tumors. Mutations in the *STK11* gene were more frequent in cases harboring *KRAS* and *BRAF* mutations. No significant differences were seen within actionable gene subclasses.

Distinctive genes and/or pathways were affected by the concurrent alterations identified in the different actionable gene subgroups, including TP53, STK11, genes associated with cycline pathways and RTKs (Figure 1 and Figure 3). Of note, TP53 mutations occurred more frequently in cases with EGFR and ERBB2 mutations compared to KRAS mutant tumors (62.9 vs. 23%, OR: 3.72, 95% CI 1.99–7.10, *p* = 0.00047 and 100 vs. 23%, RR: 3.21, 95% CI 2.50–4.13, *p* = 0.031, respectively) and to ALK-rearranged cases (62.9 vs. 26%, OR: 4.52, 95% CI 1.94–11.15 and 100 vs. 26.8%, RR 3.73, 95% CI 2.25–6.18, *p* = 0.003, respectively) (Figure S4A).

STK11 co-mutations, as expected, were highly represented in KRAS mutant tumors (15.6%), but were also detected in cases with BRAF (40%), ROS1 (8.3%), ALK (2.4%) and EGFR (1.6%) alterations (Figure 3). However, the likelihood to detect STK11 mutations was significantly less in EGFR, MET, ALK and RET positive tumors compared to tumors harboring KRAS and BRAF mutations, and in ROS1-positive compared to BRAF-positive cases (Figure S4B).

Co-alterations affecting cycline pathways, including CDK2/4, occurred more frequently in MET (34.5%)-positive tumors compared to KRAS mutants (8.9%), ALK (2.4%) and RET (0%) fusion-positive cases (*p* = 0.0157; *p* = 0.0031; *p* = 0.0157, respectively) and in tumors with ERBB2 mutations (50%) compared to ALK and RET-rearranged cases (*p* = 0.0473; *p* = 0.0473, respectively) (Figure S4C).

RTKs alterations were observed across all driver gene groups except for ERBB2 (Figure 3). MET-deregulated tumors had the highest rate (65.5%) of RTKs alterations, which was significantly higher compared to KRAS (22.2%), ALK (12.2%), ERBB2 (0%), RET (6.25%) and ROS1 (25%) (Figure S4D).

No statistically significant differences were observed in the incidence of co-mutations involving other pathways, including beta-catenin, MYC pathway, PI3K pathway and DNA repair pathways.

## 4. Discussion

An increasing amount of data are emerging on genomic profiling of NSCLC through NGS platforms [20,25,26]. Previous reports selectively described potentially relevant co-altered genes in specific oncogene-addicted NSCLC subsets. The most explored are the *KRAS* and *EGFR* mutant NSCLCs, with major findings on *TP53*, *STK11* and cycline-related genes such as *CDKN2A/B* and *CDK4/6* [12,13,14,15,18,19]. The present study evaluated the incidence and patterns of concurrent genomic aberrations, across the eight main actionable genes identified in NSCLC. We found statistically significant differences in concurrent alteration profiles within the eight subgroups both quantitively and qualitatively. Indeed, we observed differences in the frequencies of co-occurrences, which were lower in *ALK*-rearranged tumors, in median co-alteration number, lower in *ALK*, *RET* and *ROS1*-rearranged tumors and higher in *MET*-deregulated cases, and in the occurrence of specific co-alteration categories (*TP53*, *STK11*, cycline-related genes and RTKs).

Previous findings in *EGFR*-mutant lung cancers show recurrent aberrations in *TP53* (54.6–64.6%), *PIK3CA* (9−12.4%), *CTNNB1* (5.3−9.6%), *CDKN2A/B* (20.2–24.6%) and *CDK4* (7−10%) [18,27,28]. Taking into account our categorization according to gene functions, the rates of these co-occurrent alterations are quite comparable with those observed in our cohort of *EGFR*-mutant tumors (*TP53* 62.9%, *PIK3CA* pathway 16.1%, cycline-related genes 17.7% and β-catenin 3.2%). The observed rate of *TP53* mutations in *ALK*, *RET* and *ROS1*-rearranged tumors (26.8, 56.2 and 41.7%, respectively) was also similar to that reported in other studies (23.8−26.5% for *ALK*, 34.6−45.5% for *RET* and 45.6% for *ROS1*) [25,29,30]. Conversely, we found a lower rate of mutations involving cycline-related genes in the three rearranged subgroups (2.4, 0 and 8.3%, in *ALK, RET* and *ROS1*, respectively) compared to the previous reports [25,29]. However, given that the analysis of gene copy number loss (e.g., *CDKN2A/B* loss) was not included in our study, these differences may be related to the types of gene alterations covered by different NGS panels. In tumors with *MET* exon 14 skipping mutations we identified a similar rate of *TP53* co-mutations and cycline-related gene alterations as compared to previous findings (33.3 and 50% in our study; 33.5 and 17.5–41.6% in historical data, respectively) [25]. Previous reports on concurrent alterations in *BRAF*-mutant lung cancer, including V600 and non-V600 alterations, showed a higher rate of *TP53* (53.3%), lower rate of *STK11* (16.2%) and a similar rate of *PIK3CA* (6.6%), *MYC* (10.8%) and *CDKN2A/B* (11.2–16.6%) alterations [25], compared to our study (*TP53* 30%, *STK11* 16.2%, *PIK3CA* pathway 10%, *MYC* pathway 10% and cyclines pathway 10%). Finally, *TP53* co-mutations were reported in 51.7%, *CDKN2A/B* in 17.2–27.2%, *PIK3CA* in 5% and *CTNNB1* in 4.4% of *ERBB2*-mutant NSCLC [25], but these data are not comparable to our results due to the very small number of tumors with *ERBB2* mutations included in our cohort.

Taken together, our data reveal a lower frequency of concurrent alterations in tumors driven by fusion genes, and higher rates of co-occurrences in those driven by other actionable gene alterations, especially *MET* amplification/mutation. These observations potentially suggest differential tumor clonal evolution among the actionable gene groups [31]. Indeed, a more linear evolutionary model might be distinctive of gene fusion-driven tumors, with gene rearrangements occurring as very early events in clonal evolution, in the absence of relevant co-occurring mutations. Conversely, the higher rate of co-occurrent alterations, either “passengers” or even “drivers”, in *MET*, *KRAS*, *BRAF* and *EGFR*-driven tumors, suggests a branched clonal evolutionary process existing from the early steps of tumor progression. The presence of multiple subclones that may compete during tumor progression and, more importantly, during anti-cancer treatments, can drive the establishment of drug-tolerant persister clones and subsequent off-target drug resistance [7,8,32]. Hence, the specific patterns and relative abundance of co-occurring alterations might be responsible for differential tumor behavior in terms of response to anti-cancer treatments, duration of response and treatment resistance mechanisms. In the evolving experience of the failure of mono-immunotherapy in the main oncogene-addicted NSCLCs, the deeper investigation of co-alteration profiles could shed light on potential subgroups that might still benefit from such an approach or require combinatorial strategies [33,34,35].

The significance and relative impact of co-occurring gene alterations are strongly dependent on several factors, including the size of the evaluated cohort and the number and type of evaluated gene alterations according to the specific sequencing platform. However, to our knowledge, this is the first in-class study to comprehensively describe and categorize co-mutation patterns according to primary actionable gene alterations in advanced NSCLC, providing an additional exploratory comparison among subgroups. The relatively high rate of actionable gene alterations—including those of rarer genes—observed in our study is most likely due to the referral nature of many patients, which results in a positive selection of cases with a higher probability to be found with driver alterations (e.g., never-smokers with previously diagnosed negative *EGFR* mutational status accessing our institution for wider tumor genomic profiling). However, the absence of previous oncogene-driven treatments for the advanced setting in the evaluated samples cohort is a major strength in our study, since it allows more accurate interpretation of co-mutation patterns, that might be influenced by selective pressure induced by prior therapies. The main limitation of this study is the unavailability of clinical data, due to the relatively short follow-up time of the included population, the non-homogeneous nature of treatment administered in relation to the heterogeneous populations and the referral nature of many patients’ samples who were treated in other institutions.

## 5. Conclusions

The treatment scenario for patients with NSCLC has dramatically changed in the last two decades thanks to the advent of different targeted agents and immune checkpoint inhibitors. The use of these agents significantly improved the median survival benefit in the advanced setting. However, different open questions remain to further improve the survival of patients with NSCLC and improve our understanding of non-response or resistance in patients with oncogene-addicted NSCLC, in which the role of concurrent alterations could be crucial. Indeed, the analysis of co-occurring gene alterations might help in clinical practice, by guiding personalized treatment selection and resistance evaluation. However, despite being strongly recommended, NGS is still not currently standard in many institutions; therefore, the clinical applicability in the real-world setting of investigating co-occurring mutations remains for now in the field of translational research. Based on the exploratory results of the current study, further investigation is planned to selectively collect clinical data in each actionable gene subgroup, defining homogeneously treated patient populations, and to identify differential co-occurrence patterns to be correlated with treatment outcomes, resistance patterns and survival.

## Figures and Tables

**Table 1:** Frequencies of co-mutation and concurrent copy number gain variants in the eight actionable gene groups.

Driver Gene Mutation	*n* (%)	Presence of Co-Mutations (%)		Presence of CNVs (%)	
*KRAS* G12C non-G12C	135 (43.7%) 49 (36.3%) 86 (63.7%)	104 (77%) 37 (75.5%) 67 (77.9%)	*p* value = 0.75	51 (37.8%) 20 (40.8%) 31 (36%)	*p* value = 0.58
*EGFR* del19 + L858R other	62 (20%) 50 (80.6%) 12 (19.4%)	52 (83.9%) 41 (82%) 11 (91.7%)	*p* value = 0.41	36 (58.1%) 31 (62%) 5 (41.7%)	*p* value = 0.19
*MET* exon14 skipping amplification (CNV ≥ 6)	29 (9.4%) 6 (20.7%) 23 (79.3%)	25 (86.2%) 2 (33.3%) 23 (100%)	*p* value < 0.001	14 (48.3%) 4 (66.7%) 10 (43.5%)	*p* value = 0.08
*BRAF* V600 non-V600	10 (3.2%) 3 (30%) 7 (70%)	9 (90%) 2 (66.7%) 7 (100%)	*p* value = 0.65	14 (48.3%) 4 (66.7%) 10 (43.5%)	*p* value = 0.08
*ALK*	41 (13.3%)	18 (43.9%)		5 (12.2%)	
*HER2*	4 (1.3%)	4 (100%)		2 (50%)	
*RET*	16 (5.2%)	11 (68.8%)		3 (18.8%)	
*ROS1*	12 (3.9%)	7 (58.3%)		1 (8.3%)	
Total	309 (100%)	229 (74.1%)		114 (36.9%)	

## Data Availability

The data presented in this study are available in insert article and supplementary materials.

## Supplementary Materials

The following are available online at https://www.mdpi.com/article/10.3390/cancers13092172/s1, Figure S1: Distribution of concurrent actionable gene alterations, Figure S2: Differences in the median number of concurrent alterations of the eight actionable gene groups, Figure S3: Differences in frequency of co-mutation (A) and copy number gain variants (B) of the eight actionable gene groups, Figure S4: Differences in the frequency of *TP53* (A) and *STK11* (B) co-mutations, cycline pathways (C) and RTKs (D) co-alterations of the eight actionable gene groups, Table S1: Genes included in the nine concurrent alteration categories, Table S2: Main characteristics of included population, File S1. Co-occurrence tendency of the eight actionable gene according to the mutual exclusivity analysis from the cBioPortal Oncoprinter (https://www.cbioportal.org/oncoprinter, accessed on 10 March 2021).
