# Supplementary material for: Genomic Characterization of Concurrent Alterations in Non-Small Cell Lung Cancer (NSCLC) Harboring Actionable Mutations
Source: Cancers (Basel). 2021 Apr 30;13(9):2172. doi: 10.3390/cancers13092172 (PMC8124171; doi:10.3390/cancers13092172)
Supplement: Supplementary file 1 [file cancers-13-02172-s001.zip › Supplementary Figures.pdf]

cancers

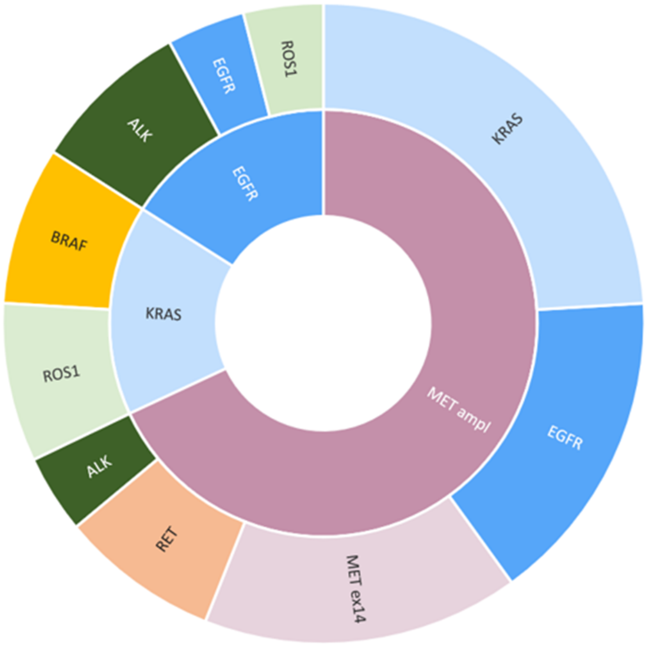

Figure S1. Distribution of concurrent actionable gene alterations.

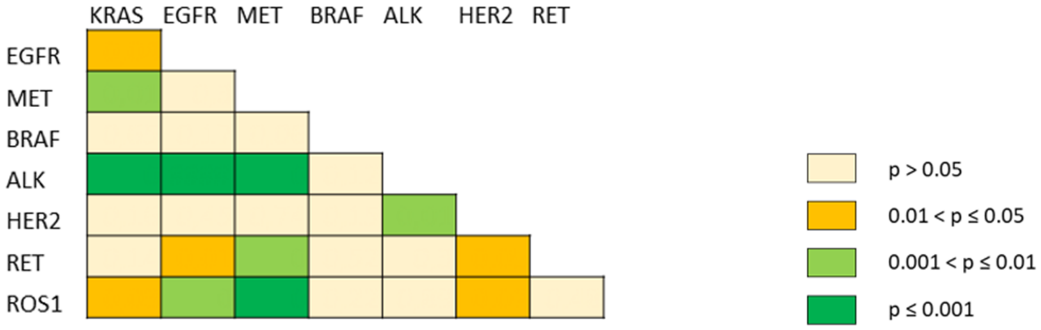

Figure S2. Differences in the median number of concurrent alterations of the eight actionable gene groups. Statistical significance was evaluated according to pairwise comparisons using t-tests with pooled standard deviation (SD) and color-coded according to the legend.

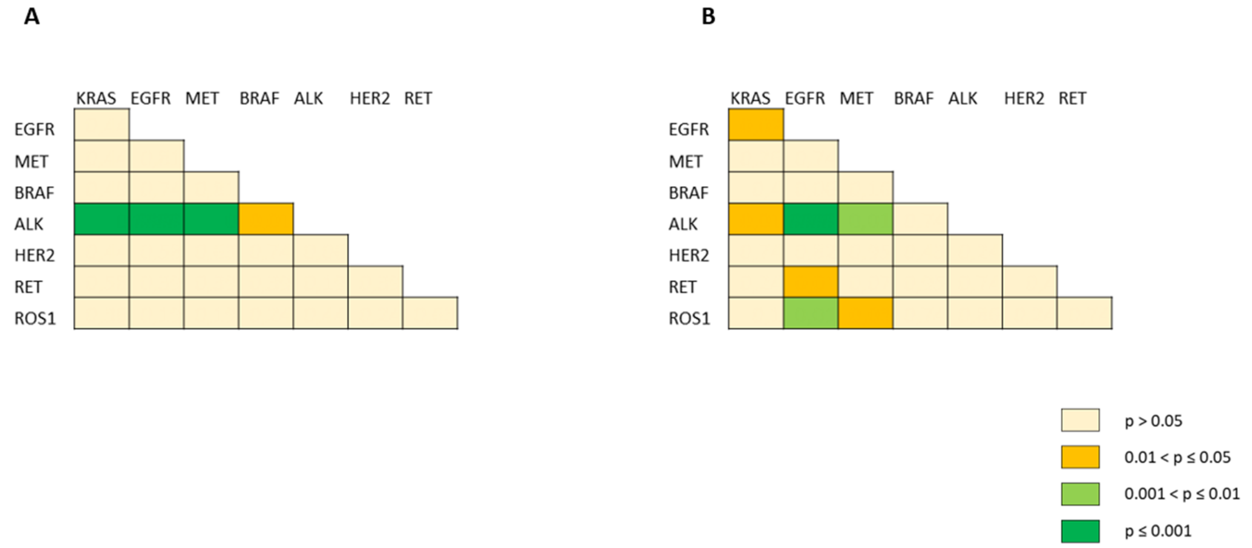

Figure S3: Differences in frequency of co-mutation (**A**) and copy number gain variants (**B**) of the eight actionable gene groups. Statistical significance was evaluated according to pairwise comparisons using t-tests with pooled standard deviation (SD) and color-coded according to the legend.

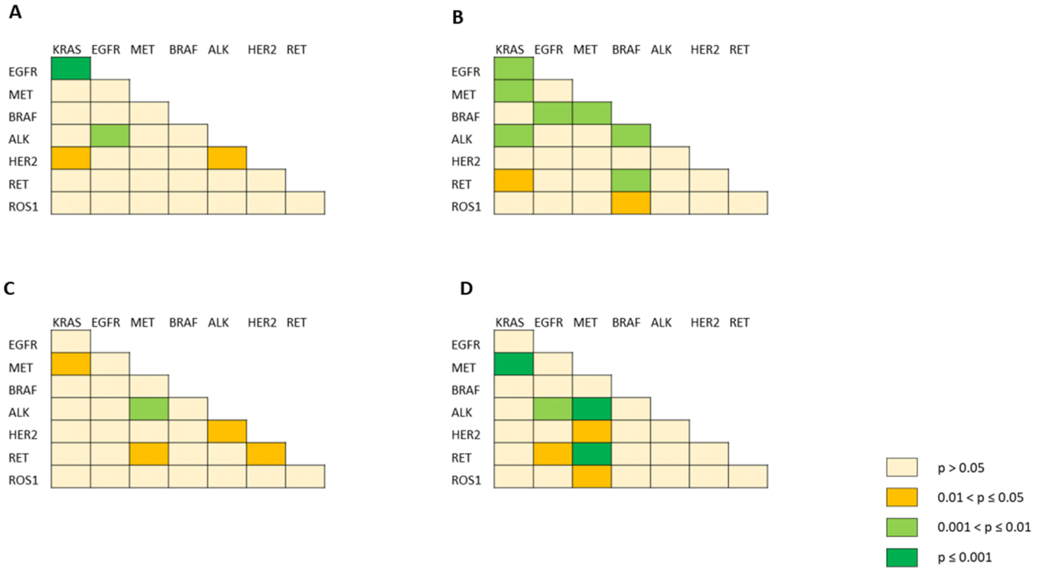

Figure S4. Differences in the frequency of *TP53* (**A**) and *STK11* (**B**) co-mutations, cycline pathways (**C**) and RTKs (**D**) co-alterations of the eight actionable gene groups. Statistical significance was evaluated according to pairwise comparisons using t-tests with pooled standard deviation (SD) and color-coded according to the legend.

Table S1. Genes included in the nine concurrent alteration categories.

Co-Mutation category	Included Genes *
TP53	*TP53*
STK11	*STK11*
MYC	*MYC, MYCL, MYCN, MAX, PTEN*
PI3K	*PIK3CA, PIK3R1, PIK3CB, AKT1, AKT2, AKT3, MTOR, RICTOR*
DNA repair	*BRCA1, BRCA2, PALB2, RAD50, RAD51B, ATM, ATR, ATRX, MLH1, MSH6*
Beta-catenin	*CTNNB1*
Cyclines pathway	*CDKN2A, CDKN2B, CCND1, CCND3, CCNE1, CDK4, CDK2, CDK6*
RTKs	*EGFR, KRAS, MET, ALK, BRAF, ROS1, RET, ERBB2, ERBB3, KIT, PDG-FRA, FGFR1, FGFR2, NTRK1, NTRK3, TRK3*

Others	*PITCH1, ARID1A, SETD2, RNF43, SMAD4, NOTCH1, NOTCH2, NOTCH3, IDH1, IDH2, NF1, NF2, RB1, CREBBP, SMARCA4, U2AF1, HNF1A, HIST1H3B, EIF3E, FANCA, ESR1, SMO, JAK1, JAK3, NFE2L2, MDM2, MDM4, TERT, AR, TSC2, FBXW7, FGF19, FGF3*

*point mutations, copy number variants, gene rearrangements.

Table 2. Main characteristics of included population.

**Clinical Characteristics**	***N* (%)**
Sex	
Male	159 (56)
Female	125 (44)
Age, median (range)	62 (29-89)
Smoking status	
Current	88 (31)
Former	128 (45)
Never	68 (24)
Tissue type	
Primary lung/regional lymph nodes	205 (72)
Distant metastasis	79 (28)
Collection method	
Tissue block	204 (72)
Cytoblock/cytological slides	80 (28)

File S1. Co-occurrence tendency of the eight actionable gene according to the mutual exclusivity analysis from the cBioPortal Oncoprinter (http:// https://www.cbioportal.org/oncoprinter).
